# A small shift, a major leap: Changing gender‐role attitudes among adolescents across two ethnic groups

**DOI:** 10.1002/jad.12393

**Published:** 2024-08-18

**Authors:** Suha Daw, Miri Scharf

**Affiliations:** ^1^ Department of Counseling and Human Development University of Haifa Haifa Israel

**Keywords:** adolescence, ethnic differences, gender‐role attitudes, growth mindset intervention, sexism

## Abstract

**Introduction:**

The current study examined a growth mindset intervention designed to promote egalitarian gender role attitudes among adolescents during a pivotal stage of their development, as these attitudes may have important implications for their identity development, well‐being, and future life decisions.

**Methods:**

A sample of 181 eighth‐grade students (61% female, *M*_age_ = 13.14, SD = 0.42) from six Israeli schools participated in the study. The sample consisted of 49% Jewish and 51% Arab adolescents, including both Muslims and Christians. Adolescents engaged in a two‐session intervention that included videos and reflective writing tasks. Pre‐and postintervention, they completed self‐administered questionnaires assessing their gender‐role mindsets, attitudes toward women, and sexism. The data collection and intervention process took place from late 2021 to early 2023.

**Results:**

After the intervention, there was an increase in growth mindsets and egalitarian attitudes towards women among adolescents, and a reduction in benevolent sexism and fixed gender‐role mindsets. Hostile sexism, however, remained unchanged. No significant sex or ethnic differences were found in the effectiveness of the intervention. Gender‐role mindsets mediated the association between the intervention and egalitarian attitudes, but not the association between the intervention and benevolent sexism.

**Conclusions:**

The findings demonstrate the potential of brief and targeted growth mindset interventions in promoting favorable changes adolescents' attitudes towards gender roles. According to this study, despite prolonged gender‐role socialization, adolescents from diverse ethnic backgrounds can move towards more egalitarian attitudes and flexibility in gender roles through a rather targeted process. This finding is promising especially in adolescence, when stereotypes are often intensified.

## Graphical abstract

This study evaluated the effectiveness of an intervention aimed at fostering a growth mindset—the belief that abilities can develop through effort—in adolescents' gender‐role attitudes across two ethnic groups. Findings show increased growth mindsets egalitarian attitudes towards women, decreased fixed mindsets, and benevolent sexism, but no change in hostile sexism. The findings suggest that adolescents can shift towards more equal gender role attitudes through a focused intervention, despite prolonged gender‐role socialization, and the intensification of gender stereotypes during adolescence.

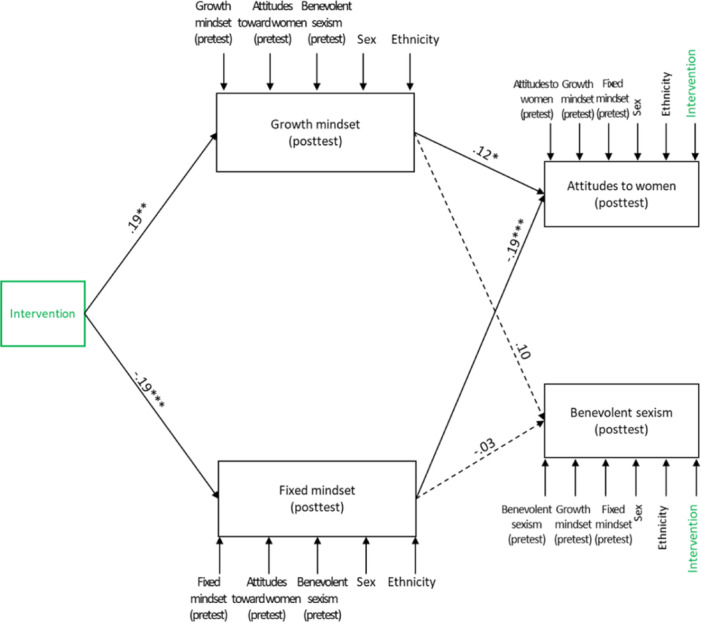

## 1 INTRODUCTION

Adolescence is characterized by significant physical, psychological, cognitive, and social transformations (Lerner & Steinberg, 2004). One of the central tasks during this time is identity formation (Erikson, 1994), including shaping gender‐role attitudes regarding the rights, roles, and responsibilities of men and women in the public and private spheres (Côté, 2009; Gupta & Santhya, 2020). Puberty accentuates biological gender differences and cognitive advances, such as abstract thinking and reasoning, that enables more profound self‐reflection and exploration of gender identity (Branje et al., 2021; Steensma et al., 2013). Moreover, the emergence of dating and sexual interest during adolescence can contribute to gender intensification (Korlat et al., 2022; Klaczynski et al, 2020). According to gender intensification theory (Hill & Lynch, 1983), adolescents face increased pressure to conform to culturally sanctioned gender roles, which leads boys to act in more stereotypically masculine ways and girls to act in more stereotypically feminine ways. Research indicates that during these formative years, adolescents' gender‐role attitudes increasingly reflect those of their close social networks, including parents and friends, underscoring the significant influence of their immediate social environment on their gender identity (Sánchez Guerrero et al., 2018).

Social factors might perpetuate traditional and unequal gender roles (Wood & Eagly, 2015), as well as encourage sexist attitudes (King et al., 2019). According to the Ambivalent Sexism Theory (Glick & Fiske, 1996, 2001), sexist attitudes refer to beliefs, stereotypes, and prejudices that discriminate against individuals based on their gender. Individuals with hostile sexist attitudes perceive women's sexual power as threatening to men, and have negative attitudes toward women who violate traditional gender roles (Glick & Fiske, 1996; Ramiro‐Sánchez et al., 2018). Benevolent sexist attitudes reinforce the women's needs for men's protection, the complementary nature of women's qualities to men's, and the centrality of heterosexual intimacy for achieving happiness in relationships (Ferguson & Donnellan, 2017).

Numerous studies indicated that adolescents' internalization of traditional and sexist gender‐role attitudes can be related to their behavior and overall well‐being (Halimi et al., 2021; King et al., 2019; Moreau et al., 2021; Ward & Grower, 2020). These attitudes might limit adolescents' aspirations (Brown, 2019) and affect their academic and career choices in a way that might not reflect their abilities and interests (Leaper & Brown, 2018; Wong et al., 2017). Furthermore, more traditional attitudes were associated with lower levels of intimacy, self‐disclosure, and respect in interpersonal relationships (Giaccardi et al., 2017; Horne & Johnson, 2018).

Societal norms towards gender roles can vary greatly across different ethnic contexts, with some favoring traditional and sexist attitudes, while others promote more egalitarian approaches toward gender roles (Basu et al., 2017; Oakley, 2016). This difference is often influenced by the level of individualism and secularism in a culture, with more individualistic and secular societies generally supporting more equal gender roles, while collectivist and religious societies tend to uphold more traditional and sexist attitudes (Hofstede, 1980; Kalmijn, 2003). Furthermore, gender norms within these contexts may differ based on biological sex, with males typically endorsing more traditional gender‐role attitudes that grant them greater autonomy and freedom, while females generally seek increased autonomy and tend to embrace more egalitarian attitudes (Gesser‐Edelsburg & Arabia, 2018; Nayak et al., 2003). Immigrant and nonimmigrant adolescents in Germany exhibited distinct trajectories in their gender role attitudes (Wilhelm et al., 2023). Males from traditional backgrounds reported more conventional and unequal gender‐role attitudes compared to others. These findings underscore the influence of both ethnic background and biological sex on adolescents' gender attitudes.

Societal roles assigned to men and women can be perceived as either fixed or malleable (Kray et al., 2017). Individuals with fixed gender‐role mindset associate specific traits solely with one gender, reinforcing traditional gender stereotypes. In contrast, individuals with growth gender‐role mindset view roles and behaviors as influenced by actions and circumstances, rather than being predetermined and immutable (Kray et al., 2017; Townsend et al., 2023). Fostering healthy development and promoting positive interpersonal relationships (Lowe et al., 2022; Stewart et al., 2021) are important task during adolescence. In the current study, we evaluated the effectiveness of a growth mindset intervention in promoting positive attitudes towards women and growth gender‐role mindsets, and reducing both hostile and benevolent sexism, and fixed gender‐role mindsets.

### 1.1 Gender‐role growth mindset interventions

Research indicates that adolescents may naturally develop more egalitarian gender‐role attitudes as they grow older (Sánchez Guerrero et al., 2018). Targeted interventions during this formative phase can considerably intensify and accelerate this development, thereby reinforcing and perpetuating the positive shifts in gender‐role attitudes. Moreover, the pervasive influence of internet content that often reinforces gender stereotypes highlights the need for proactive interventions to support the development of healthy gender‐role attitudes (Baams et al., 2015; Bartini, 2006; Blum, 2020; Hunersen et al., 2023; Moreau et al., 2021). Previous studies on gender‐role interventions yielded mixed findings across diverse contexts. Positive outcomes in altering gender‐role attitudes were reported in studies conducted in India (Gupta & Santhya, 2020; Santhya & Zavier, 2022), the United Kingdom (Spinner et al., 2021), and the United States (Kato‐Wallace et al., 2019). Conversely, other studies in the United States and South Africa did not show significant changes (Ralfe, 2009; Savasuk‐Luxton et al., 2018). These findings suggest that although cultural context plays a role in shaping attitudes, the effectiveness of interventions may be largely determined by their specific methodologies and implementation strategies. These strategies include using a guiding theoretical framework, direct participant education, messages emphasizing gender equality rather than highlighting disadvantages of inequalities, and the engagement of role models (Levy et al., 2020; Lowe et al., 2022; Ruane‐McAteer et al., 2020; Stewart et al., 2021). The current intervention aligns with these findings.

Our intervention was guided by the principles of the growth mindset theory (Dweck, 2012; Yeager et al., 2016). In general, growth mindset interventions have shown to successfully alter individuals' beliefs about intelligence, personality traits, motivation, and group dynamics, while generally being adaptable for school settings (Blackwell et al., 2007; Goldenberg et al., 2017; Paunesku et al., 2015; Yeager et al., 2016). These interventions, conducted in a single session or across multiple sessions (Burgasser, 2019; DeBacker et al., 2018; Rattan et al., 2015; Yeager & Dweck, 2012), often utilize educational materials, such as videos or articles, to illustrate the flexibility and changeability of human abilities, and the potential for growth through persistent effort (Babinski et al., 2018; Schleider & Weisz, 2018; Yeager et al., 2013). Although research specific to gender‐role growth mindset interventions is limited, studies with adult populations demonstrated their effectiveness in reducing stereotypical endorsement (Carr et al., 2012; Halperin et al., 2011, 2012; Levontin et al., 2013; Levy et al., 1998) and gender stereotyping (Kray et al., 2017; Townsend et al., 2023).

### 1.2 The current study

The current study examines the effectiveness of gender‐role growth mindset interventions, aimed at encouraging positive attitudes towards women, and reducing sexist attitudes. Previous studies conducted with American adults (Kray et al., 2017; Townsend et al., 2023) suggested potential benefits. However, there is a need to examine such interventions in adolescence, especially among adolescents from diverse cultural contexts.

This study was conducted among adolescents residing in Israel, representing a multicultural context with a Jewish majority (74%) and an Arab minority (21%) (Central Bureau of Statistics, 2022). Israeli society has witnessed substantial changes in gender relations over past decades, particularly in women's participation in the workforce and their educational attainment and occupational status (Mandel & Birgier, 2016). These developments have been driven by feminist activism and supportive legislative and institutional frameworks, which aimed to promote gender equality (Chazan, 2018).

The pace and extent of these changes vary across different segments of Israeli society. Within the Jewish majority, secular communities generally hold more egalitarian attitudes on gender roles, with greater acceptance of women's participation in the public domain, and a more equal division of household responsibilities (Mandel & Birgier, 2016). On the other hand, the Arab minority, although experiencing modernization, continues to hold more traditional and patriarchal norms (Abu‐Rabia‐Queder, 2017). Notably, Arab society is described as a “society in transition,” with women increasingly participating in public and economic domains (Sabbah‐Karkaby & Stier, 2017). However, these changes are gradual and occur alongside ongoing adherence to traditional values (Vitman‐Schorr & Ayalon, 2020). Consequently, Arab adolescents, in particular, often face greater pressure to conform to traditional gender expectations related to modesty, family responsibilities, and limited public participation (Malka et al., 2021; Vitman‐Schorr & Ayalon, 2020). This setting provides a unique context in which to test the effectiveness of the intervention.

We hypothesized that the intervention would result in a positive shift in attitudes within the intervention group, characterized by heightened egalitarian attitudes toward women and growth gender‐role mindsets, as well as reduced hostile and benevolent sexist attitudes, and a decline in fixed gender‐role mindsets. Additionally, we explored whether changes in gender‐role mindsets mediated the effect of the intervention on adolescents' attitudes. We hypothesized that the intervention would elevate growth mindset levels and attenuate fixed mindset regarding gender roles, and, in turn, enhance egalitarian attitudes toward women and decrease hostile and benevolent sexism among adolescents.

## 2 MATERIALS AND METHODS

### 2.1 Participants

Participants were 181 eighth‐grade students, with a female majority (61%), an age range of 12–14 years old, and a mean age of 13.14 years (SD = 0.42). The participants were drawn from six public schools located in northern Israel. We specifically chose public schools, which account for 57.2% of all schools in Israel, for our study, to better reflect the general school population and ensure that our findings are broadly applicable. In the sample, 49% were Jewish and 51% Arab, comprised of 35% Muslims and 16% Christians. Most participants (72%) reported average economic status. Mothers of 46% of the participants had an academic education and 27% reported the same for their fathers.

Of the 181 students who participated at baseline, 24% (*n* = 43) did not participate in the posttest. The dropout rate was influenced by irregular school attendance during the coronavirus disease 2019 (COVID‐19) pandemic, and difficulties matching questionnaires, as some students did not consistently use the required four‐digit password. Power analysis was conducted for the remaining *n* = 138 participants, using G*Power (Faul et al., 2009). Assuming an average effect size of the group by time interaction (*f* = 0.25) and *α* of .05, power was 0.87. Thus, the current study has adequate power.

Missing data analysis using Little's MCAR test indicates a missing at random pattern (*χ* ^2^(27) = 42.24, *p* < .05). Follow up tests indicate that no differences were found in the attrition rates from the intervention and comparison groups (*χ* ^2^(1) = 0.42, *p* = .52), nor were there differences associated with cultural affiliation (*χ* ^*2*^(1) = 0.16, *p* = .69). Attrition was associated with gender (*χ* ^2^(1) = 9.01, *p* < .01), indicating higher dropout of boys (36% boys vs. 16% girls). A series of *t* tests indicated that those who dropped out had lower egalitarian attitudes toward women (dropouts *M* = 2.93, SD = 0.66; retained *M* = 3.23, SD = 0.55, *t* = 2.92, *p* < .01) and lower gender‐role growth mindset (dropouts *M* = 3.38, SD = 1.25; retained *M* = 4.21, SD = 1.24; *t* = 3.79, *p* < .001). No significant differences were revealed for hostile sexism (*t* = 1.63, *p* = .11), benevolent sexism (*t* = 0.03, *p* = .98), and gender‐role fixed mindset (*t* = 0.45, *p* = .66).

## 3 PROCEDURE

Ethical approval was obtained from the University Faculty Ethics Committee and the Ministry of Education, before the study. Data collection and the intervention took place between December 2021 and January 2023. Due to the challenges posed by the COVID‐19 pandemic, particularly the disruption of school routines, it was difficult to obtain agreement from school principals to include more than one class per school in the study. This practical constraint led us to seek two additional schools in which to establish the comparison group. We used the cultivation index (CI), a widely accepted measure used by the Ministry of Education in Israel, to minimize potential differences between the groups from different schools, as well as similarities in school profiles published on the Ministry of Education's website, and include several school climate criteria, as further detailed on the description of background information at "Measures" section. As presented in Table 1, we did not find significant differences between participants assigned to the intervention or comparison groups in terms of sociodemographic characteristics.

**Table 1:** Sample characteristics.

Variable	Intervention (*n* = 121)	Comparison (*n* = 60)		*p*
Participant's age (*M*, SD)	13.10 (0.38)	13.02 (1.83)	*t* (175) = 0.08	.65
Participant's sex (%)				
Male	*n* = 45 (37.2%)	*n* = 25 (41.7%)	$\chi^{2}(1)=0.34$	.56
Female	*n* = 76 (62.8%)	*n* = 35 (58.3%)		
Participant's ethnic affiliation				
Jewish (%)	*n* = 59 (48.8%)	*n* = 30 (50%)	$\chi^{2}(1)=0.03$	.88
Arab (%)	*n* = 62 (51.2%)	*n* = 30 (50%)		
Family income				
Below average	*n* = 5 (4.4%)	*n* = 4 (6.8%)	$\chi^{2}(2)=0.68$	.71
Average	*n* = 88 (77.9%)	*n* = 43 (72.9%)		
Above average	*n* = 20 (17.7%)	*n* = 12 (20.3%)		
Mother's education				
High school graduate	*n* = 40 (35.4%)	*n* = 21 (35.6%)	$\chi^{2}(2)=1.69$	.64
Vocational certificate	*n* = 20 (17.7%)	*n* = 11 (18.6%)		
Bachelor degree or higher	*n* = 35 (46.9%)	*n* = 27 (45.8%)		
Father's education				
High school graduate	*n* = 48 (43.2%)	*n* = 23 (39.6%)	$\chi^{2}(2)=3.41$	.33
Vocational certificate	*n* = 32 (28.8%)	*n* = 20 (34.5%)		
Bachelor degree or higher	*n* = 31 (27.9%)	*n* = 15 (25.9%)		

All questionnaires were translated into Hebrew and Arabic by three languages experts, and final versions were created based on discussions and consensus on disagreements. Before their participation, adolescents and their parents gave written consent. The study was conducted for both intervention and comparison groups in a classroom setting, under the supervision of the principal researcher and a research assistant, and in the presence of the class teacher. In the initial phase of the study adolescents completed an online questionnaire on their mobile devices, using Qualtrics. This process was repeated 1 month later for both groups, with participants receiving a coupon for ice cream upon completion of the questionnaire as an incentive. For the intervention group, the initial phase was followed by a two‐session intervention, with a week between sessions. Each session lasted ~40 min and included watching a video and completing a reflective writing task. No supplementary activities were conducted in the comparison group during the 1‐month gap between visits.

Due to the unpredictable conditions of the COVID‐19 pandemic, such as recurring school closures and inconsistent student attendance, we set a 1‐month interval for the follow‐up to ensure and enhance students' participation. We chose to conduct the follow‐up in person to maintain consistency with the initial intervention and posttest, and to encourage increased engagement and reduced dropout rates in the classroom setting. We thought that a planned session in the classroom would minimize dropout, as students would be more likely to attend and participate in a structured, in‐person setting. However, we acknowledge that this choice limited our ability to assess long‐term changes in attitudes.

### 3.1 Intervention

The content of the intervention was based on empirical research findings on implicit theories, particularly with regard to group stereotyping (Levontin et al., 2013; Levy et al., 1998; Rydell et al., 2007), and gender role stereotypes (Kray et al., 2017). The content of the intervention included explicit messages that emphasized the malleability of gender roles and the advantages of gender equality, such as broader prospects for personal and professional development, enhanced cooperation and problem‐solving between genders, and healthier cross‐gender relationships founded on mutual respect and equitable responsibilities. The intervention also integrated media critical literacy strategies to enhance participants' awareness and analysis of traditional gender roles and stereotypes in media content (Gordon et al., 2020; Xie et al., 2019).

The first session started with a video that highlighted fixed and growth mindsets regarding gender roles, the capacity of the brain to change, the potential for personal advancement through effort and support, and the notion that biological sex does not dictate specific traits, characteristics, or interests. The video featured popular figures from diverse fields who challenge conventional gender stereotypes. These included inspiring examples such as Kathrine Johnson, a pioneering mathematician at NASA; Mikhail Baryshnikov, a celebrated male ballet dancer; and Amelia Earhart, a groundbreaking female aviator. These role models were incorporated to illustrate the success and fulfillment achievable when one transcends traditional gender expectations. Afterwards, participants were asked to engage in a reflective writing task, during which they shared their thoughts and impressions of the video.

The second session built upon the first by presenting a video that summarized the key themes of the initial session, and presented the negative consequences of non‐egalitarian attitudes in relationships. The video also aimed to enhance media literacy skills by examining the portrayal and perpetuation of gender roles in various forms of media. Following the video, participants were instructed to compose a letter to younger students, summarizing the key messages and providing examples and advice for critical media consumption.

### 3.2 Measures

#### 3.2.1 Gender‐role mindsets

A 10‐item scale assessing the perceived immutability/flexibility of gender roles, in which five items measured gender‐role fixed mindsets (e.g., “I think that men and women will always have different social roles), and five items measured gender‐role growth mindsets (e.g., “Both men and women are well‐suited for most societal roles”). A 6‐point Likert scale was used ranging from “*strongly disagree*” to “*strongly agree*,” with higher scores indicating greater endorsement of the belief that gender roles are immutable/flexible. Cronbach's *α* for fixed and growth mindset were .61 and .69, respectively, in the pre‐intervention condition, and .80 and .73, respectively, in the postintervention condition.

#### 3.2.2 Attitudes toward women

Twelve items from the 15‐item short version of the Attitudes Toward Women Scale (Spence & Helmreich, 1978) were employed to assess attitudes toward the rights and roles of women (e.g., “Women should assume their rightful place in business and all professions along with men”). A 4‐point Likert scale was used ranging from “*totally disagree*” to “*totally agree*.” Higher scores reflect more egalitarian attitudes toward women. The Cronbach *α* in both the pre‐ and postintervention conditions was .83.

#### 3.2.3 Sexist attitudes

The short version (12‐items) of the Ambivalent Sexism Inventory (Glick and Whitehead, 2010) was employed to examine hostile sexism (e.g., “Women seek to gain power by getting control over men”), and benevolent sexism (e.g., “Women should be cherished and protected by men”). A 6‐point Likert scale was used ranging from “*totally disagree*” to “*totally agree*.” Higher scores reflect greater endorsement of hostile and benevolent sexism. Cronbach's *α* for hostile and benevolent sexism were .74 and .62, respectively in the pre‐intervention condition, while they were .83 and .68, respectively in the postintervention condition.

#### 3.2.4 Background information

Respondents were asked to provide sociodemographic information about their age, religion, socioeconomic status (SES), and number of siblings. To determine the SES of the participants, we utilized the five‐category index created by the Israeli Ministry of Education for schools. This index is based on a combination of four factors: education of the most educated parent (40%), level of income per capita in the family (20%), peripherality of the settlement (20%), and a combination of immigration and family origin (whether from a distressed country) (20%). We randomly selected schools classified as CI level 3, which represents a mid‐tier SES. This approach intended to maximize the generalizability of the study by avoiding extremes of high or low SES. Additionally, participants were also asked to evaluate their family's economic status and the educational levels of their parents.

## 4 RESULTS

### 4.1 Sex and ethnic differences

The results indicated some significant effects for sex and ethnic affiliation, which justified controlling for these. As presented in Table 2, males displayed significantly higher fixed gender‐role mindset (*M* = 4.08, SD = 1.21) than females (*M* = 3.43, SD = 1.26), females displayed significantly higher growth gender‐role mindset (*M* = 3.67, SD = 1.30) than males (*M* = 4.57, SD = 1.13). Moreover, males displayed significantly fewer egalitarian attitudes towards women (*M* = 2.89, SD = 0.64) than females (*M* = 3.44, SD = 0.42), and significantly more hostile sexism (*M* = 3.72, SD = 1.09) than females (*M* = 2.79, SD = 0.97). We did not find significant sex differences in benevolent sexism between males (*M* = 3.93, SD = 0.92) and females (*M* = 4.18, SD = 0.98).

**Table 2:** Results of repeated measure ANCOVA analyses of intervention versus comparison group on gender‐role mindsets and attitudes while controlling sex and ethnic affiliation.

	Group *M* (SD)		Within‐subject effects				Between‐subject effects		
Measure	Intervention *n* = 94	Comparison *n* = 44	*F* (1,134) time (${ղ}^{2}$)	*F* (1,134) time × ethnicity (${ղ}^{2}$)	*F* (1,134) time × sex (${ղ}^{2}$)	*F* (1,134) time × group (${ղ}^{2}$)	*F* (1,134) ethnicity (${ղ}^{2}$)	*F* (1,134) sex (${ղ}^{2}$)	*F* (1,134) group (${ղ}^{2}$)
Attitudes toward women									
Pre	3.20 (0.59)	3.30 (0.49)	2.79	0.14	1.03	38.32***	27.94***	44.76***	0.02
Post	3.41 (0.55)	3.23 (0.49)	(0.02)	(0.00)	(0.01)	(0.22)	(0.17)	(0.25)	(0.00)
Hostile sexism									
Pre	3.03 (1.05)	3.06 (0.86)	1.05	0.75	0.15	0.07	5.95*	35.90***	0.00
Post	3.09 (1.17)	3.18 (1.06)	(0.01)	(0.01)	(0.00)	(0.00)	(0.04)	(0.21)	(0.00)
Benevolent sexism									
Pre	4.23 (0.99)	4.02 (0.85)	0.25	0.59	1.03	12.31***	6.55*	3.90	0.19
Post	3.89 (1.03)	4.20 (0.86)	(0.00)	(0.00)	(0.01)	(0.08)	(0.05)	(0.03)	(0.00)
Growth gender‐role mindset									
Pre	4.19 (1.32)	4.20 (1.08)	1.97	0.09	0.53	6.99**	5.13*	25.17***	0.95
Post	4.71 (1.22)	4.24 (1.02)	(0.02)	(0.00)	(0.00)	(0.05)	(0.04)	(0.16)	(0.01)
Fixed gender‐role mindset									
Pre	3.85 (1.28)	3.96 (1.00)	2.26	4.10*	3.21	7.40**	0.02	11.97***	2.24
Post	3.22 (1.42)	3.80 (1.13)	(0.02)	(0.03)	(0.02)	(0.05)	(0.00)	(0.08)	(0.02)

Abbreviation: ANCOVA, analysis of covariance.

* *p* < .05.

** *p* < .01.

*** *p* < .001.

With regard to ethnic differences, Jewish participants displayed significantly higher growth gender‐role mindset (*M* = 4.40, SD = 1.21) compared to Arab participants (*M* = 4.10, SD = 1.31), while we did not find ethnic differences for fixed gender‐role mindset. Moreover, Jewish participants displayed more egalitarian attitudes towards women (*M* = 3.45, SD = 0.47) than Arabs (*M* = 3.04, SD = 0.61), more hostile sexism (*M* = 3.26, SD = 1.14) than Arabs (*M* = 2.99, SD = 1.05), and less benevolent sexism (*M* = 3.90, SD = 0.97) than Arabs (*M* = 4.28, SD = 0.92).

Results also indicate a significant ethnic affiliation (2) × time (2) interaction with regard to fixed mindset. When probing the interaction, we found that Jewish (*M* = 3.81, SD = 1.15) and Arab (*M* = 3.97, SD = 1.23) participants did not significantly differ at baseline (*F*_1,131_ = 0.44, *p* = .510, *η*_*p*_^2^ = 0.00). However, Arab participants displayed a more significant decrease in pre–post fixed gender‐role mindset (*M* = 3.48, SD = 1.29, *F*_1,131_ = 33.07, *p* < .001, *η*_*p*_^2^ = 0.20), whereas Jewish participants displayed a less, but still significant, pre–post decrease (*M* = 3.33, SD = 1.42, *F*_1,131_ = 8.50, *p* = .004, *η*_*p*_^2^ = 0.06).

### 4.2 The effects of the intervention on gender‐role mindsets and attitudes

To test the effects of the intervention on fixed and growth gender‐role mindsets, attitudes towards women, hostile and benevolent sexism, we employed the IBM SPSS 27 to perform a series of two‐way repeated analysis of covariance (ANCOVA) measures with group (intervention vs. comparison) as a between‐subject factor and time (pre vs. post) as a repeated measures factor, while controlling for sex and ethnic affiliation. Table 2 presents the between and within‐subject effects in the intervention and comparison groups across time, as well as descriptive statistics for both groups and pre/post‐intervention. As seen in Table 2, we did not find any main effects for time or group. However, the group (2) × time (2) repeated‐measures ANCOVA indicated significant interaction effects between time and group for fixed and growth gender‐role mindsets, attitudes towards women, and benevolent sexism, although not for hostile sexism.

When probing the interaction for the dependent variables, we found that the intervention and comparison groups did not significantly differ at pre‐intervention for fixed (*F*_1,131_ = 0.15, *p* = .71, *η*_*p*_^2^ = 0.00) and growth mindsets (*F*_1,130_ = 0.08, *p* = .78, *η*_*p*_^2^ = 0.00), attitudes towards women (*F*_1,134_ = 2.20, *p* = .14, *η*_*p*_^2^ = 0.01), and benevolent sexism (*F*_1,134_ = 1.35, *p* = .25, *η*_*p*_^2^ = 0.01). However, in line with the hypotheses, in the intervention group, fixed gender‐role mindset decreased following the intervention (*F*_1,131_ = 43.31, *p* < .001, *η*_*p*_^2^ = 0.25), while for the comparison group no significant change was found between pre‐ and postmeasurements (*F*_1,131_ = 1.59, *p* = .21, *η*_*p*_^2^ = 0.01). Growth gender‐role mindset significantly increased following the intervention (*F*_1,130_ = 25.17, *p* < .001, *η*_*p*_^2^ = 0.16), while for the comparison groups no significant change was found between pre and postmeasurements (*F*_1,130_ = 0.08, *p* = .78, *η*_*p*_^2^ = 0.00). In addition, attitudes towards women were more egalitarian following the intervention (*F*_1,134_ = 68.69, *p* < .001, *η*_*p*_^2^ = 0.34), while for the comparison groups no significant change was found between pre‐ and postmeasurements (*F*_1,134_ = 3.37, *p* = .07, *η*_*p*_^2^ = 0.03). Similarly, for benevolent sexism, we found a decrease in the intervention group from pre to post (*F*_1,134_ = 16.10, *p* < .001, *η*_*p*_^2^ = 0.11), while for the comparison groups no significant change in benevolent sexism was found between measurements (*F*_1,134_ = 2.28, *p* = .13, *η*_*p*_^2^ = 0.02).

**Figure 1:**
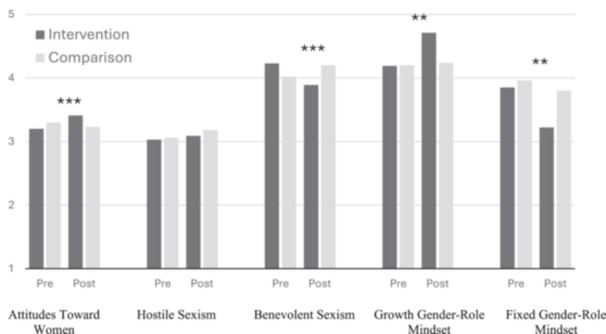
Effects of the intervention on gender‐role mindsets and attitudes. **p < .01, ***p < .001.

As presented in Figure 1, the findings indicate that following the intervention, growth mindset increased, while fixed mindset decreased. The findings also suggest that attitudes towards women became more egalitarian and benevolent sexism decreased following the intervention. No change was found for hostile sexism.

### 4.3 Robustness analyses

Given that the findings indicate a consistent intervention by time interaction, we thus focused on the extent to which these interaction effects might be moderated by sex and ethnicity. To examine the moderation effects of sex and ethnicity, we conducted a series of repeated ANCOVA measures in which we included triple interactions: sex × group × time and ethnicity × group × time. The results indicate that the intervention effects reported earlier are quite robust to sex and ethnic affiliation. For detailed results of these analyses, see Supporting Information S1: Appendix A. We did, however, find ethnicity as a moderator for attitudes toward women (*F*_1,133_ = 6.22, *p* = .014, *η*_*p*_^2^ = 0.045). Probing the interaction revealed that the intervention effect was more profound among Arab participants (*F*_1,67_ = 31.64, *p* < .001, *η*_*p*_^2^ = 0.321) than Jewish participants (*F*_1,67_ = 9.09, *p* = .004, *η*_*p*_^2^ = 0.12).

### 4.4 Mediational analysis

Given that the intervention found to significantly affect adolescents' mindset and gender role attitudes, we further examined whether gender‐role mindset could mediate the effect of the intervention on gender‐role attitudes. To do so, we followed Valente and MacKinnon's (2017) recommendation and applied structural equation modeling (SEM) to estimate a mediation effect in a pre–posttest comparison group design. In this research design, the experimental manipulation (*X*) assumes to mark the beginning of the mediational chain. Mediation effect is estimated as the mediated effect of the intervention (*X*) effect on posttest gender‐role attitudes (Y2) through its effect on the posttest mediator mindset (M2). The adjustment of pretest measures of the mediator (M1) and the outcome (Y1), as well as the cross‐lagged effects, allow the M2–Y2 relation to be free of any time‐invariant third variable effects, and be considered a causal effect. In line with previous findings, we controlled for sex and ethnicity effects in all analyses. Analysis was performed with IBM AMOS 27 (Arbuckle, 2020).

**Figure 2:**
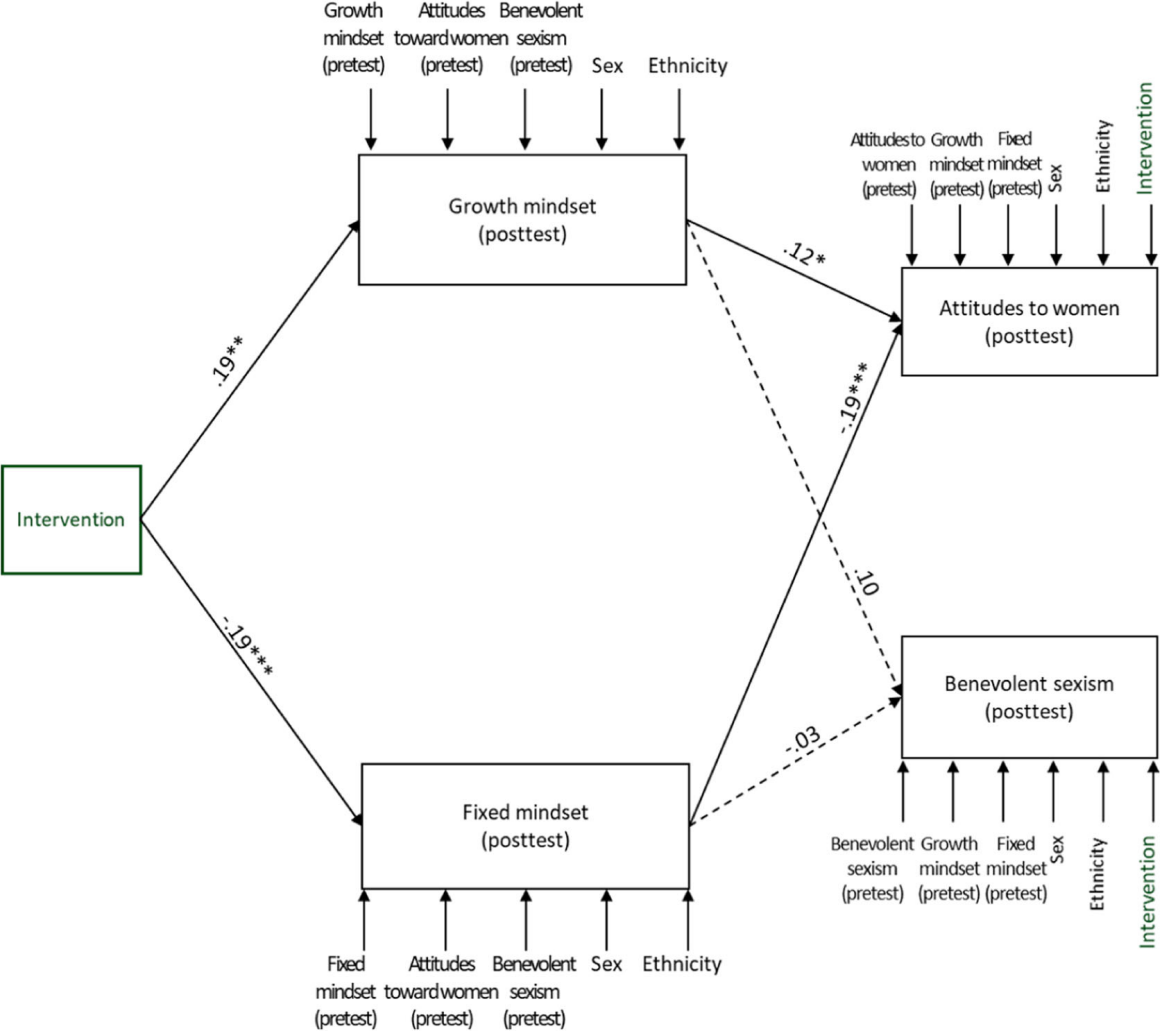
The effect of the intervention on gender‐role attitudes through gender‐role mindset: A mediation model in a pre‐ posttest comparison design. For reasons of visual clarity, only *a* and *b* paths are presented. Other effects in the model are presented as short arrows, their effects can be found in Table 2. Full lines represent significant effects and broken lines represent nonsignificant effects. *p < .05, **p < .01, ***p < .001.

The SEM pre–posttest comparison group model showed adequate fit indices (*χ* ^2^ = 10.71, *df* = 4, *p* = .03, normed fit index = 0.99, comparative fit index = 0.99, root mean square error of approximation = 0.11). Standardized path coefficients are presented in Table 3. Given the complexity of the model, a simplified visual representation of the results is presented in Figure 2. As can be seen, and in line with the repeated ANCOVA measures, the intervention enhanced gender‐role growth mindset and reduced gender‐role fixed mindset that, in turn, significantly influenced egalitarian attitudes toward women. The significance of the mediation effect was then estimated using 95% CI of 5000 bootstrap samples. Results support the mediation effects for both growth mindset (indirect *β* = .02, *B* = 0.03, 95% CI = 0.003, 0.06) and fixed mindset (indirect *β* = .03, *B* = 0.04, 95% CI = 0.02, 0.08). With regard to benevolent sexism, growth mindset and fixed mindset did not significantly affect benevolent sexism, and test of mediation indicates that the effect of the intervention on benevolent sexism does not occur through growth mindset (indirect *β* = .02, *B* = 0.04, 95% CI = −0.03, 0.13) or fixed mindset (indirect *β* = .005, *B* = 0.02, 95% CI = −0.061, 0.121).

**Table 3:** Mediation analysis in pre–posttest comparison group design: An SEM model.

		Mediators				Outcomes			
		Postgrowth mindset		Postfixed mindset		Post‐attitudes toward woman		Postbenevolent sexism	
		*Β*	*P*	*β*	*p*	*β*	*p*	*β*	*p*
Intervention	*a* path	**0.19**	**.002**	**−.19**	**<.001**	.15	<.001	‐.24	<.001
Pregrowth mindset		0.54	<.001	–	–	−.08	.114	‐.13	.170
Prefixed mindset		–	–	.63	<.001	.08	.109	.04	.694
Prebenevolent sexism		0.07	.243	−.03	.595	–	–	.61	<.001
Pre‐attitudes toward women		0.19	.022	−.21	.002	.77	<.001	–	–
Sex		0.05	.469	.05	.382	.04	.367	.13	.082
Ethnicity		−0.05	.424	−.17	.003	−.12	.002	.02	.739
Postgrowth mindset	*b* path	–	–	–	–	**.12**	**.015**	**.10**	**.278**
Postfixed mindset	*b* path	–	–	–	–	**−.19**	**<.001**	**−.03**	**.739**

*Note*: Intervention: 0 = comparison, 1 = intervention; Sex: 0 = male, 1 = female; Ethnicity: 0 = Jewish, 1 = Arab. The mediation *a* paths and *b* paths are bold.

Abbreviation: SEM, structural equation modeling.

## 5 DISCUSSION

### 5.1 Sex and ethnic differences

Before delving into the main findings of the study, it is important to recognize the differences that we observed in gender‐role mindsets and attitudes of adolescents based on their biological sex and ethnicity, as they provide a framework for the results of our study. The differences between males and females, as well as between Jewish and Arab adolescents, were observed in the majority of gender‐role mindsets and attitudes, and may be attributed to the socialization of sociocultural norms, which may influence gender‐role attitudes during the formative stage of adolescence (Carter, 2014). In particular, male adolescents exhibited fewer growth mindsets and fewer egalitarian attitudes towards women than females. Moreover, they exhibited more fixed mindsets and higher levels of hostile sexism than females. These findings align with previous research and may be attributed to societal pressures that encourage adolescent males to conform to traditional masculine gender roles (Landry et al., 2020; Skewes et al., 2021). Although male and female adolescents may differ in their openness to gender equality, our findings indicate that they exhibit similar levels of benevolent sexism. This might be attributed to a widespread tendency and social norms that often idealize protective male behaviors, as reflected in benevolent sexism (Baker, 2010; Hammond et al., 2018).

The study also revealed ethnic differences in gender‐role mindsets and attitudes, with Jewish adolescents exhibiting more growth mindsets and egalitarian attitudes towards women compared to Arab adolescents. These differences could potentially be linked to the different cultural norms prevalent in their societies. Secular Jewish society, which often tends to be more individualistic and supportive of gender equality, may differ from Arab society, which often adheres to more traditional norms (Gesser‐Edelsburg & Arabia, 2018; Obeid et al., 2010).

However, Jewish adolescents reported higher levels of hostile sexism than Arab adolescents. Previous research indicated that in societies that tend to prioritize individualism, young people frequently view social relationships as competitive in nature, a characteristic that aligns with hostile sexism, which emphasizes the need to prove one's worth, and the potential struggle for resources. This might lead to feeling pressure to conform to certain gender norms to gain acceptance (Hofstede, 2011; Lee et al., 2007). Conversely, Arab adolescents displayed more benevolent sexism, potentially aligning with collectivist values that emphasize interdependence and maintenance of harmonious relations despite power disparities (de Lemus et al., 2010; Lee et al., 2007). Nevertheless, both Jewish and Arab adolescents exhibited similar levels of fixed gender‐role mindsets. This might be indicative of profound social and historical factors that extend beyond particular cultural contexts, pointing to a more generalized pattern in the development of gender role perceptions. Further cross‐cultural research could explain the origins and potential modifications of these attitudes and gender socialization practices.

### 5.2 The effects of the intervention

The study findings reveal that as a result of the intervention, there was an increase in adolescents' growth gender‐role mindsets and egalitarian attitudes towards women, and a decrease in fixed mindsets and benevolent sexism. This is noteworthy given the extended process of gender‐role socialization that shapes attitudes throughout an individual's lifetime (Carter, 2014), particularly in societies where traditional gender roles are strongly emphasized (Obeid et al., 2010). Our findings align with previous research conducted with adult populations (Kray et al., 2017; Townsend et al., 2023) and indicate that brief and focused mindset interventions may effectively encourage more egalitarian attitudes and gender‐role flexibility. This is particularly relevant given adolescents' tendency to intensify gender stereotypes (Ramiro‐Sánchez et al., 2018). Moreover, such changes in gender‐role attitudes may impact adolescents' immediate educational and social development, as well as their long‐term relationships and aspirations (Moreau et al., 2021).

However, contrary to studies that documented reductions following longer interventions (Carrascosa et al., 2019; Navarro‐Pérez et al., 2020), the intervention did not result in a decrease in hostile sexism. Hostile sexism, characterized by active opposition or aggression towards women, may necessitate more in‐depth, sustained intervention strategies to address deeply ingrained prejudices (de Lemus et al., 2010; Glick & Fiske, 2001; Zell et al., 2016). The limited duration and scope of our brief intervention may not have been sufficient to challenge these entrenched attitudes, indicating a need for longer‐term, comprehensive approaches (Sanz‐Barbero et al., 2022).

The findings further revealed that the positive impact of the growth mindset intervention was found among adolescents of both genders and both ethnic backgrounds. This suggests the potential generalizability of the intervention to individuals from various demographics. However, further investigation across different ethnic backgrounds is required to establish its universality. A moderating effect was observed in the attitudes towards women, where Arab participants exhibited greater change in these attitudes compared to Jewish participants. This promising result suggests that Arab participants, who initially held more traditional attitudes toward women than Jewish participants, might have had more room for change and benefit from the intervention, despite their initial attitudes.

Moreover, the association between the intervention and egalitarian attitudes towards women was mediated by increased growth gender‐role mindsets and decreased fixed gender‐role mindsets. This finding suggests that promoting beliefs about the malleability of gender roles among adolescents could be a promising strategy that potentially leads to an increase in support for gender equality. By emphasizing the idea that gender roles can be changed and developed, adolescents may be more inclined to challenge and question existing gender norms and stereotypes, resulting in a more equitable perspective (Malespina et al., 2022; Yu et al., 2021).

The impact of the intervention on reducing benevolent sexism was not mediated by changes in gender‐role mindsets. Benevolent sexism, often disguised as positive or protective attitudes, might require specific intervention components such as media literacy, and exposure to nontraditional role models to address its subtle, romanticized aspects and challenge the idealized gender role notions therein (Sanz‑Barbero et al., 2022).

### 5.3 Limitations and future directions

The study was conducted during the COVID‐19 pandemic, which posed challenges in recruiting schools and reaching a broader participant base. Additionally, the irregular school attendance due to health guidelines probably contributed to the higher dropout rate. Notably, adolescents who did not participate in the second assessment reported lower levels of egalitarian attitudes towards women than other participants. They possibly encountered varying circumstances that affected their continued engagement, or lacked the motivation to continue participation. Future research could benefit from employing strategies to enhance participant retention in more stable conditions, such as conducting a preparation process at the school, using reminders, and monitoring continuous participation. Moreover, selecting intervention and comparison groups from different schools presents a limitation that may affect the results observed. Although we attempted to match the schools based on CI and school profile information, there may be unobserved differences between the schools, such as educational resources, school norms, or peer influences that might impact gender role attitudes. Future studies might consider sampling both groups from the same school population to better control for these environmental factors. Additionally, there were activities as part of the intervention, during which the intervention groups and the experimenters interacted. The comparison groups interacted with experimenters only when completing the questionnaires. To control possible influence of these interactions, future studies should also include some sort of guided interaction with the experimenters. Although our study yielded significant findings, the 1‐month follow‐up might not have been sufficient to evaluate long‐lasting changes in attitudes. Future studies should incorporate extended follow‐up periods to better understand the sustainability of intervention impacts, and to provide valuable insights into the long‐term effects of such interventions. Finally, integrating qualitative methods with self‐report questionnaires would provide a deeper understanding of participants' attitudes and could help mitigate potential biases in self‐reported data.

In conclusion, this study represents a preliminary investigation into the potential of growth mindset interventions in changing gender attitudes in the crucial developmental period of adolescence. The results demonstrate the effectiveness of the intervention in promoting egalitarian gender‐role attitudes among adolescents across both genders and ethnic groups. This underlines the suitability of the intervention for educational and social settings, showing that even short interventions can contribute significantly to changing gender‐role attitudes during this formative stage, to promote and sustain balanced gender‐role attitudes among adolescents.

## CONFLICT OF INTEREST STATEMENT

We have no known conflict of interest to disclose.

## ETHICS STATEMENT

The study received ethical approval from the University Faculty Ethics Committee and the Chief Scientist Bureau in the Ministry of Education. Active parental consent for adolescents' participation was obtained from all participants. This research was not preregistered.

## Supporting information

Supporting Information

## Data Availability

The data used in the research cannot be publicly shared, but are available upon request from the corresponding author. Restrictions apply to the availability of these data due to the sensitive nature of the topic and the confidentiality of the participants.

## References

[jad12393-bib-0001] Abu‐Rabia‐Queder, S. (2017). The paradox of professional marginality among Arab‐Bedouin women. Sociology, 51(5), 1084–1100. 10.1177/0038038516641621

[jad12393-bib-0002] Arbuckle, J. L. (2020). IBM SPSS Amos 27 user's guide (pp. 226–229). Amos Development Corporation, SPSS Inc. https://www.csun.edu/itr/downloads/docs/IBM_SPSS_Amos_User_GuideV23.pdf

[jad12393-bib-0003] Baams, L. , Dubas, J. S. , Overbeek, G. , & Van Aken, M. A. G. (2015). Transitions in body and behavior: A meta‐analytic study on the relationship between pubertal development and adolescent sexual behavior. Journal of Adolescent Health, 56(6), 586–598. 10.1016/j.jadohealth.2014.11.019 25636818

[jad12393-bib-0004] Babinski, L. M. , Murray, D. W. , Wilson, W. A. , Kuhn, C. M. , & Malone, P. S. (2018). Impact of a neuroscience‐based health education course on high school students' health knowledge, beliefs, and behaviors. Journal of Adolescent Health, 63(4), 489–496. 10.1016/j.jadohealth.2018.05.016 30286902

[jad12393-bib-0005] Baker, J. (2010). Claiming volition and evading victimhood: Post‐feminist obligations for young women. Feminism & Psychology, 20(2), 186–204. 10.1177/0959353509359142

[jad12393-bib-0006] Bartini, M. (2006). Gender role flexibility in early adolescence: Developmental change in attitudes, self‐perceptions, and behaviors. Sex Roles, 55(3–4), 233–245. 10.1007/s11199-006-9076-1

[jad12393-bib-0403] Basu, S. , Zuo, X. , Lou, C. , Acharya, R. , & Lundgren, R. (2017). Learning to be gendered: Gender socialization in early adolescence among urban poor in Delhi, India, and Shanghai, China. Journal of Adolescent Health, 61(4), S24–S29. 10.1016/j.jadohealth.2017.03.012 28915988

[jad12393-bib-0007] Blackwell, L. S. , Trzesniewski, K. H. , & Dweck, C. S. (2007). Implicit theories of intelligence predict achievement across an adolescent transition: A longitudinal study and an intervention. Child Development, 78(1), 246–263. 10.1111/j.1467-8624.2007.00995.x 17328703

[jad12393-bib-0008] Blum, R. W. (2020). Gender norm transformative programing: Where are we now? where do we need to be? Journal of Adolescent Health, 66(2), 135–136. 10.1016/j.jadohealth.2019.11.299 31952565

[jad12393-bib-0009] Branje, S. , De Moor, E. L. , Spitzer, J. , & Becht, A. I. (2021). Dynamics of identity development in adolescence: A decade in review. Journal of Research on Adolescence, 31(4), 908–927. 10.1111/jora.12678 34820948 PMC9298910

[jad12393-bib-0010] Brown, C. S. (2019). Sexualized gender stereotypes predict girls' academic self‐efficacy and motivation across middle school. International Journal of Behavioral Development, 43(6), 523–529. 10.1177/01650254198623

[jad12393-bib-0011] Burgasser, A. J. (2019). Why I teach growth mindset. Nature Astronomy, 3(12), 1038–1040. 10.1038/s41550-019-0940-7

[jad12393-bib-0012] Carr, P. B. , Rattan, A. , & Dweck, C. S. (2012). Implicit theories shape intergroup relations. Advances in Experimental Social Psychology, 45, 127–165. 10.1016/B978-0-12-394286-9.00003-2

[jad12393-bib-0013] Carrascosa, L. , Cava, M. J. , Buelga, S. , & de Jesus, S. N. (2019). Reduction of sexist attitudes, romantic myths, and aggressive behaviors in adolescents: Efficacy of the DARSI program. Psicothema, 31(2), 121–127. 10.7334/psicothema2018.245 31013235

[jad12393-bib-0014] Carter, M. (2014). Gender socialization and identity theory. Social Sciences, 3(2), 242–263. 10.3390/socsci3020242

[jad12393-bib-0015] Central Bureau of Statistics . (2022). Statistical Abstract of Israel (Vol. 2022, p. 73).

[jad12393-bib-0016] Chazan, C. N. (2018). Israel at 70: A gender perspective. Israel Studies, 23(3), 141–151.

[jad12393-bib-0017] Côté, J. E. (2009). Identity formation and self‐development in adolescence. In R. M. Lerner , & L. Steinberg (Eds.), Handbook of adolescent psychology (pp. 266–304). Wiley & Sons.

[jad12393-bib-0018] DeBacker, T. K. , Heddy, B. C. , Kershen, J. L. , Crowson, H. M. , Looney, K. , & Goldman, J. A. (2018). Effects of a one‐shot growth mindset intervention on beliefs about intelligence and achievement goals. Educational Psychology, 38(6), 711–733. 10.1080/01443410.2018.1426833

[jad12393-bib-0019] Dweck, C. S. (2012). Mindsets and human nature: Promoting change in the Middle East, the schoolyard, the racial divide, and willpower. American Psychologist, 67(8), 614–622. 10.1037/a0029783 23163438

[jad12393-bib-0020] Erikson, E. H. (1994). Identity and the life cycle. WW Norton & Company.

[jad12393-bib-0021] Faul, F. , Erdfelder, E. , Buchner, A. , & Lang, A. G. (2009). Statistical power analyses using G*Power 3.1: Tests for correlation and regression analyses. Behavior Research Methods, 41(4), 1149–1160. 10.3758/BRM.41.4.1149 19897823

[jad12393-bib-0022] Ferguson, C. J. , & Donnellan, M. B. (2017). Are associations between “sexist” video games and decreased empathy toward women robust? A reanalysis of Gabbiadini et al. 2016. Journal of Youth and Adolescence, 46, 2446–2459. 10.1007/s10964-017-0700-x 28639206

[jad12393-bib-0023] Gesser‐Edelsburg, A. , & Arabia, M. A. E. (2018). Discourse on exposure to pornography content online between Arab adolescents and parents: Qualitative study on its impact on sexual education and behavior. Journal of Medical Internet Research, 20(10), e11667. 10.2196/11667 30305264 PMC6231764

[jad12393-bib-0024] Giaccardi, S. , Monique Ward, L. , Seabrook, R. C. , Manago, A. , & Lippman, J. R. (2017). Media use and men's risk behaviors: Examining the role of masculinity ideology. Sex Roles, 77, 581–592. 10.1007/s11199-017-0754-y

[jad12393-bib-0025] Glick, P. , & Fiske, S. T. (1996). The ambivalent sexism inventory: Differentiating hostile and benevolent sexism. Journal of Personality and Social Psychology, 70, 491–512.

[jad12393-bib-0026] Glick, P. , & Fiske, S. T. (2001). Ambivalent stereotypes as legitimizing ideologies: Differentiating paternalistic and envious prejudice. In J. T. Jost , & B. Major (Eds.), The psychology of legitimacy: Emerging perspectives on ideology, justice, and intergroup relations (pp. 278–306). Cambridge University Press.

[jad12393-bib-0401] Glick, P. , & Whitehead, J. (2010). Hostility toward men and the perceived stability of male dominance. Social Psychology, 41(3), 2151–2590. 10.1027/1864-9335/a000025

[jad12393-bib-0027] Goldenberg, A. , Endevelt, K. , Ran, S. , Dweck, C. S. , Gross, J. J. , & Halperin, E. (2017). Making intergroup contact more fruitful: Enhancing cooperation between Palestinian and Jewish‐Israeli adolescents by fostering beliefs about group malleability. Social Psychological and Personality Science, 8(1), 3–10. 10.1177/1948550616672851

[jad12393-bib-0402] Gordon, C. S. , Rodgers, R. F. , Slater, A. E. , McLean, S. A. , Jarman, H. K. , & Paxton, S. J. (2020). A cluster randomized controlled trial of the SoMe social media literacy body image and wellbeing program for adolescent boys and girls: Study protocol. Body Image, 33, 27–37. 10.1016/j.bodyim.2020.02.003 32086189

[jad12393-bib-0028] Gupta, A. K. , & Santhya, K. G. (2020). Promoting gender egalitarian norms and practices among boys in rural India: The relative effect of intervening in early and late adolescence. Journal of Adolescent Health, 66(2), 157–165. 10.1016/j.jadohealth.2019.03.007 31227386

[jad12393-bib-0029] Halimi, M. , Davis, S. N. , & Consuegra, E. (2021). The power of peers? Early adolescent gender typicality, peer relations, and gender role attitudes in Belgium. Gender Issues, 38, 210–237. 10.1007/s12147-020-09262-3

[jad12393-bib-0030] Halperin, E. , Crisp, R. J. , Husnu, S. , Trzesniewski, K. H. , Dweck, C. S. , & Gross, J. J. (2012). Promoting intergroup contact by changing beliefs: Group malleability, intergroup anxiety, and contact motivation. Emotion (Washington, D.C.), 12(6), 1192–1195. 10.1037/a0028620 22642339

[jad12393-bib-0031] Halperin, E. , Russell, A. G. , Trzesniewski, K. H. , Gross, J. J. , & Dweck, C. S. (2011). Promoting the Middle East peace process by changing beliefs about group malleability. Science, 333, 1767–1769. 10.1126/science.1202925 21868627

[jad12393-bib-0032] Hammond, M. D. , Milojev, P. , Huang, Y. , & Sibley, C. G. (2018). Benevolent sexism and hostile sexism across the ages. Social Psychological and Personality Science, 9(7), 863–874. 10.1177/1948550617727588

[jad12393-bib-0033] Hill, J. P. , & Lynch, M. E. (1983). The intensification of gender‐related role expectations during early adolescence. In J. Brooks‐Gunn , & A. C. Petersen (Eds.), Girls at puberty: Biological and psychosocial perspectives (pp. 201–228). Springer US. 10.1007/978-1-4899-0354-9_10

[jad12393-bib-0034] Hofstede, G. (1980). Culture and organizations. International Studies of Management & Organization, 10(4), 15–41.

[jad12393-bib-0035] Hofstede, G. (2011). Dimensionalizing cultures: The Hofstede model in context. Online Readings in Psychology and Culture, 2(1), 8. 10.9707/2307-0919.1014

[jad12393-bib-0036] Horne, R. M. , & Johnson, M. D. (2018). Gender role attitudes, relationship efficacy, and self‐disclosure in intimate relationships. The Journal of Social Psychology, 158(1), 37–50. 10.1080/00224545.2017.1297288 28375758

[jad12393-bib-0037] Hunersen, K. , Li, M. , Pinandari, A. W. , Mbela, P. , van Reeuwijk, M. , Barker, K. M. , Maddaleno, M. , & Moreau, C. (2023). Understanding how gender transformative interventions affect adolescent sexuality: A cross‐cultural perspective. Journal of Adolescent Health, 73(1), S65–S73. 10.1016/j.jadohealth.2023.02.030 37330823

[jad12393-bib-0038] Kalmijn, M. (2003). Country differences in sex‐role attitudes: Cultural and economic explanations. In W. Arts , J. Hagenaars , L. Halman , W. Van De Donk , & T. Schaik , The cultural diversity of European unity: Findings, explanations, and reflections from the European values study (pp. 311–337). Brill.

[jad12393-bib-0039] Kato‐Wallace, J. , Barker, G. , Garg, A. , Feliz, N. , Levack, A. , Ports, K. , & Miller, E. (2019). Adapting a global gender‐transformative violence prevention program for the US community‐based setting for work with young men. Global Social Welfare, 6, 121–130. 10.1007/s40609-018-00135-y 30956935 PMC6444362

[jad12393-bib-0040] King, T. L. , Singh, A. , & Milner, A. (2019). Associations between gender‐role attitudes and mental health outcomes in a nationally representative sample of Australian adolescents. Journal of Adolescent Health, 65(1), 72–78. 10.1016/j.jadohealth.2019.01.011 30833116

[jad12393-bib-0404] Klaczynski, P. A. , Felmban, W. S. , & Kole, J. (2020). Gender intensification and gender generalization biases in pre‐adolescents, adolescents, and emerging adults. British Journal of Developmental Psychology, 38(3), 415–433. 10.1111/bjdp.12326 32115730

[jad12393-bib-0041] Korlat, S. , Foerst, N. M. , Schultes, M. T. , Schober, B. , Spiel, C. , & Kollmayer, M. (2022). Gender role identity and gender intensification: Agency and communion in adolescents' spontaneous self‐descriptions. European Journal of Developmental Psychology, 19(1), 64–88. 10.1080/17405629.2020.1865143

[jad12393-bib-0042] Kray, L. J. , Howland, L. , Russell, A. G. , & Jackman, L. M. (2017). The effects of implicit gender role theories on gender system justification: Fixed beliefs strengthen masculinity to preserve the status quo. Journal of Personality and Social Psychology, 112(1), 98–115. 10.1037/pspp0000124 28032774

[jad12393-bib-0043] Landry, M. , Vyas, A. , Malhotra, G. , & Nagaraj, N. (2020). Adolescents' development of gender equity attitudes in India. International Journal of Adolescence and Youth, 25(1), 94–103.

[jad12393-bib-0044] Leaper, C. , & Brown, C. S. (2018). Sexism in childhood and adolescence: Recent trends and advances in research. Child Development Perspectives, 12(1), 10–15. 10.1111/cdep.12247

[jad12393-bib-0045] Lee, I. C. , Pratto, F. , & Li, M. C. (2007). Social relationships and sexism in the United States and Taiwan. Journal of Cross‐Cultural Psychology, 38(5), 595–612. 10.1177/0022022107305241

[jad12393-bib-0046] de Lemus, S. , Moya, M. , & Glick, P. (2010). When contact correlates with prejudice: Adolescents' romantic relationship experience predicts greater benevolent sexism in boys and hostile sexism in girls. Sex Roles, 63, 214–225. 10.1007/s11199-010-9786-2

[jad12393-bib-0047] Lerner, R. M. , & Steinberg, L. D. (Eds.). (2004). Handbook of adolescent psychology (2nd ed). John Wiley & Sons.

[jad12393-bib-0048] Levontin, L. , Halperin, E. , & Dweck, C. S. (2013). Implicit theories block negative attributions about a longstanding adversary: The case of Israelis and Arabs. Journal of Experimental Social Psychology, 49(4), 670–675. 10.1016/j.jesp.2013.02.002

[jad12393-bib-0049] Levy, J. K. , Darmstadt, G. L. , Ashby, C. , Quandt, M. , Halsey, E. , Nagar, A. , & Greene, M. E. (2020). Characteristics of successful programmes targeting gender inequality and restrictive gender norms for the health and wellbeing of children, adolescents, and young adults: A systematic review. The Lancet Global Health, 8(2), e225–e236. 10.1016/S2214-109X(19)30495-4 31879212 PMC7025324

[jad12393-bib-0050] Levy, S. R. , Stroessner, S. J. , & Dweck, C. S. (1998). Stereotype formation and endorsement: The role of implicit theories. Journal of Personality and Social Psychology, 74(6), 1421–1436.

[jad12393-bib-0051] Lowe, H. , Dobbin, J. , Kiss, L. , Mak, J. , Mannell, J. , Watson, D. , & Devakumar, D. (2022). Mechanisms for the prevention of adolescent intimate partner violence: A realist review of interventions in low‐and middle‐income countries. PLOS Global Public Health, 2(11), e0001230. 10.1371/journal.pgph.0001230 36962608 PMC10022317

[jad12393-bib-0052] Malespina, A. , Schunn, C. D. , & Singh, C. (2022). Whose ability and growth matter? Gender, mindset and performance in physics. International Journal of STEM Education, 9(1), 28–44. 10.1186/s40594-022-00342-2

[jad12393-bib-0053] Malka, M. , Haj‐Yahia, M. M. , Sokar, S. , & Hassan‐Abbas, N. (2021). Beliefs of social work students in Israel about wife‐beating: Are they influenced by their patriarchal ideology and exposure to violence in their families‐of‐origin? Victims & Offenders, 17(2), 258–283. 10.1080/15564886.2021.1898507

[jad12393-bib-0054] Mandel, H. , & Birgier, D. P. (2016). The gender revolution in Israel: Progress and stagnation. In N. Khattab , S. Miaari , & H. Stier (Eds.), Socioeconomic inequality in Israel: A theoretical and empirical analysis (pp. 153–184). Palgrave Macmillan US.

[jad12393-bib-0055] Moreau, C. , Li, M. , Ahmed, S. , Zuo, X. , & Cislaghi, B. (2021). Assessing the spectrum of gender norms perceptions in early adolescence: A cross‐cultural analysis of the Global Early Adolescent Study. Journal of Adolescent Health, 69(1), S16–S22. 10.1016/j.jadohealth.2021.03.010 34217454

[jad12393-bib-0056] Navarro‐Pérez, J. J. , Oliver, A. , Carbonell, Á. , & Schneider, B. H. (2020). Effectiveness of a mobile app intervention to prevent dating violence in residential child care. Psychosocial Intervention, 29(2), 59–66. 10.5093/pi2020a3

[jad12393-bib-0057] Nayak, M. B. , Byrne, C. A. , Martin, M. K. , & Abraham, A. G. (2003). Attitudes toward violence against women: A cross‐nation study. Sex Roles, 49, 333–342. 10.1023/A:1025108103617

[jad12393-bib-0405] Oakley, A. (2016). Sex, gender and society. Routledge

[jad12393-bib-0058] Obeid, N. , Chang, D. F. , & Ginges, J. (2010). Beliefs about wife beating: An exploratory study with Lebanese students. Violence against women, 16(6), 691–712. 10.1177/1077801210370465 20445079

[jad12393-bib-0059] Paunesku, D. , Walton, G. M. , Romero, C. , Smith, E. N. , Yeager, D. S. , & Dweck, C. S. (2015). Mind‐set interventions are a scalable treatment for academic underachievement. Psychological Science, 26(6), 784–793. 10.1177/0956797615571017 25862544

[jad12393-bib-0060] Ralfe, E. (2009). Policy: Powerful or pointless? An exploration of the role of critical literacy in challenging and changing gender stereotypes. Language Learning Journal, 37(3), 305–321. 10.1080/09571730903208470

[jad12393-bib-0061] Ramiro‐Sánchez, T. , Ramiro, M. T. , Bermúdez, M. P. , & Buela‐Casal, G. (2018). Sexism and sexual risk behavior in adolescents: Gender differences. International Journal of Clinical and Health Psychology, 18(3), 245–253. 10.1016/j.ijchp.2018.04.002 30487930 PMC6224861

[jad12393-bib-0062] Rattan, A. , Savani, K. , Chugh, D. , & Dweck, C. S. (2015). Leveraging mindsets to promote academic achievement: Policy recommendations. Perspectives on Psychological Science, 10(6), 721–726. 10.1177/1745691615599383 26581725

[jad12393-bib-0063] Ruane‐McAteer, E. , Gillespie, K. , Amin, A. , Aventin, Á. , Robinson, M. , Hanratty, J. , Khosla, R. , & Lohan, M. (2020). Gender‐transformative programming with men and boys to improve sexual and reproductive health and rights: A systematic review of intervention studies. BMJ Global Health, 5(10), e002997. 10.1136/bmjgh-2020-002997 PMC7554509 33051283

[jad12393-bib-0406] Rydell, R. J. , Hugenberg, K. , Ray, D. , & Mackie, D. M. (2007). Implicit theories about groups and stereotyping: The role of group entitativity. Personality and Social Psychology Bulletin, 33(4), 549–558. 10.1177/01461672062969 17363758

[jad12393-bib-0064] Sabbah‐Karkaby, M. , & Stier, H. (2017). Links between education and age at marriage among Palestinian women in Israel: Changes over time. Studies in Family Planning, 48(1), 23–38. 10.1111/sifp.12015 28247945

[jad12393-bib-0407] Sánchez Guerrero, T. , Ramiro, M. T. , Bermúdez, M. P. , & Buela‐Casal, G. (2018). Sexism and sexual risk behavior in adolescents: Gender differences. International Journal of Clinical and Health Psychology, 18(3), 245–253. 30487930 10.1016/j.ijchp.2018.04.002 PMC6224861

[jad12393-bib-0065] Santhya, K. G. , & Zavier, A. J. F. (2022). Long‐term impact of exposure to a gender‐transformative program among young men: Findings from a longitudinal study in Bihar, India. Journal of Adolescent Health, 70(4), 634–642. 10.1016/j.jadohealth.2021.10.041 34952780

[jad12393-bib-0066] Sanz‐Barbero, B. , Ayala, A. , Ieracitano, F. , Rodríguez‐Blázquez, C. , Bowes, N. , De Claire, K. , Mocanu, V. , Anton‐Paduraru, D. T. , Sánchez‐SanSegundo, M. , Albaladejo‐Blázquez, N. , das Neves, A. S. A. , da Silva Queirós, A. S. , Jankowiak, B. , Waszyńska, K. , & Vives‐Cases, C. (2022). Effect of the Lights4Violence intervention on the sexism of adolescents in European countries. BMC Public Health, 22(1), 547. 10.1186/s12889-022-12925-3 35305589 PMC8933881

[jad12393-bib-0067] Savasuk‐Luxton, R. , Adler‐Baeder, F. , & Haselschwerdt, M. L. (2018). Understanding change in violence‐related attitudes for adolescents in relationship education. Journal of Adolescence, 63, 153–164. 10.1016/j.adolescence.2017.12.012 29310008

[jad12393-bib-0068] Schleider, J. , & Weisz, J. (2018). A single‐session growth mindset intervention for adolescent anxiety and depression: 9‐month outcomes of a randomized trial. Journal of Child Psychology and Psychiatry, 59(2), 160–170. 10.1111/jcpp.12811 28921523

[jad12393-bib-0069] Skewes, L. , Skewes, J. C. , & Ryan, M. K. (2021). Attitudes to sexism and the# MeToo movement at a Danish University. NORA‐Nordic Journal of Feminist and Gender Research, 29(2), 124–139. 10.1080/08038740.2021.1884598

[jad12393-bib-0408] Spence, J. T. , & Helmreich, R. (1978). Masculinity and femininity: Their psychological dimensions, correlates, and antecedents. University of Texas Press

[jad12393-bib-0070] Spinner, L. , Tenenbaum, H. R. , Cameron, L. , & Wallinheimo, A. S. (2021). A school‐based intervention to reduce gender‐stereotyping. School Psychology International, 42(4), 422–449. 10.1177/01430343211009944

[jad12393-bib-0409] Steensma, T. D. , Kreukels, B. P. , de Vries, A. L. , & Cohen‐Kettenis, P. T. (2013). Gender identity development in adolescence. Hormones and Behavior, 64(2), 288–297. 10.1016/j.yhbeh.2013.02.020 23998673

[jad12393-bib-0071] Stewart, R. , Wright, B. , Smith, L. , Roberts, S. , & Russell, N. (2021). Gendered stereotypes and norms: A systematic review of interventions designed to shift attitudes and behaviour. Heliyon, 7(4), e06660. 10.1016/j.heliyon.2021.e06660 33912699 PMC8066375

[jad12393-bib-0072] Townsend, C. H. , Kray, L. J. , & Russell, A. G. (2023). Holding the belief that gender roles can change reduces women's work–family conflict. Personality and Social Psychology Bulletin, 2(1), 8. 10.1177/01461672231178349 PMC11504165 37332232

[jad12393-bib-0073] Valente, M. J. , & MacKinnon, D. P. (2017). Comparing models of change to estimate the mediated effect in the pretest–posttest control group design. Structural Equation Modeling: A Multidisciplinary Journal, 24(3), 428–450. 10.1080/10705511.2016.1274657 28845097 PMC5568008

[jad12393-bib-0074] Vitman‐Schorr, A. , & Ayalon, L. (2020). The changing status of Israeli Arab women as reflected in their role as main caregivers. Journal of Family Issues, 41(11), 2203–2222. 10.1177/0192513X19898829

[jad12393-bib-0075] Ward, L. M. , & Grower, P. (2020). Media and the development of gender role stereotypes. Annual Review of Developmental Psychology, 2, 177–199. 10.1146/annurev-devpsych-051120-010630

[jad12393-bib-0076] Wilhelm, J. , Schober, P. S. , & Guerrero, L. S. (2023). Gender ideologies across the transition to adulthood in Germany: How early romantic relationships slow down the egalitarian trend. Advances in Life Course Research, 58, 100574. 38054876 10.1016/j.alcr.2023.100574

[jad12393-bib-0077] Wong, Y. J. , Ho, M. H. R. , Wang, S. Y. , & Miller, I. S. K. (2017). Meta‐analyses of the relationship between conformity to masculine norms and mental health‐related outcomes. Journal of Counseling Psychology, 64(1), 80–93. 10.1037/cou0000176 27869454

[jad12393-bib-0078] Wood, W. , & Eagly, A. H. (2015). Two traditions of research on gender identity. Sex Roles, 73, 461–473. 10.1007/s11199-015-0480-2

[jad12393-bib-0410] Xie, X. , Gai, X. , & Zhou, Y. (2019). A meta‐analysis of media literacy interventions for deviant behaviors. Computers & Education, 139, 146–156. 10.1016/j.compedu.2019.05.008

[jad12393-bib-0079] Yeager, D. S. , & Dweck, C. S. (2012). Mindsets that promote resilience: When students believe that personal characteristics can be developed. Educational Psychologist, 47(4), 302–314. 10.1080/00461520.2012.722805

[jad12393-bib-0080] Yeager, D. S. , Romero, C. , Paunesku, D. , Hulleman, C. S. , Schneider, B. , Hinojosa, C. , Lee, H. Y. , O'Brien, J. , Flint, K. , Roberts, A. , Trott, J. , Greene, D. , Walton, G. M. , & Dweck, C. S. (2016). Using design thinking to improve psychological interventions: The case of the growth mindset during the transition to high school. Journal of Educational Psychology, 108(3), 374–391. 10.1037/edu0000098 27524832 PMC4981081

[jad12393-bib-0411] Yeager, D. S. , Trzesniewski, K. H. , & Dweck, C. S. (2013). An implicit theories of personality intervention reduces adolescent aggression in response to victimization and exclusion. Child Development, 84(3), 970–988. 10.1111/cdev.12003 23106262 PMC3660787

[jad12393-bib-0081] Yu, J. , McLellan, R. , & Winter, L. (2021). Which boys and which girls are falling behind? Linking adolescents' gender role profiles to motivation, engagement, and achievement. Journal of Youth and Adolescence, 50, 336–352. 10.1007/s10964-020-01293-z 32734562 PMC7875942

[jad12393-bib-0082] Zell, E. , Strickhouser, J. E. , Lane, T. N. , & Teeter, S. R. (2016). Mars, Venus, or Earth? Sexism and the exaggeration of psychological gender differences. Sex Roles, 75, 287–300. 10.1007/s11199-016-0622-1

