# Supplementary material for: A small shift, a major leap: Changing gender‐role attitudes among adolescents across two ethnic groups
Source: J Adolesc. 2024 Aug 18;96(8):1942–55. doi: 10.1002/jad.12393 (PMC11618721; doi:10.1002/jad.12393)
Supplement: Supplementary file 1 — Supporting Information [file JAD-96-1942-s001.docx]

**Appendix A**

**Robustness Analyses**

Given that the findings indicated a consistent intervention by time interaction, we thus focused on the extent to which these interaction effects might be moderated by sex and ethnicity. To examine the moderation effects of sex and ethnicity we conducted a series of repeated measures ANCOVA in which we included triple interactions: sex × group × time and ethnicity × group × time.

Starting with sex, results indicated that the triple interaction sex by group by time was not significant for all the study variables: growth mindset (F_1,129_ = .46, p = .50, η_p_^2^ = .004), fixed mindset (F_1,130_ = 1.36, p = .25, η_p_^2^ = .010), attitudes toward women (F_1,133_ = .01, p = .93, η_p_^2^ = .001), hostile sexism (F_1,133_ = .05, p = .83, η_p_^2^ = .001), and benevolent sexism (F_1,133_ = .02, p = .89, η_p_^2^ = .001). These results indicate that the effect of the intervention was equivalent across sexes.

Similar findings were obtained for ethnic affiliation. The triple interaction ethnicity by group by time was not significant for growth mindset (F_1,129_ = .13, p = .71, η_p_^2^ = .001); fixed mindset (F_1,130_ = 1.09, p = .30, η_p_^2^ = .001); hostile sexism (F_1,133_ = .10, p = .75, η_p_^2^ = .001); and benevolent sexism (F_1,133_ = 1.40, p = .24, η_p_^2^ = .010). These results indicate that the effect of the intervention was equivalent across ethnic affiliation. We did however found ethnicity as a moderator for attitudes toward women (F_1,133_ = 6.22, p = .014, η_p_^2^ = .045). Probing the interaction revealed that the intervention effect was more profound for Arab participants (F_1,67_ = 31.64, p < .001, η_p_^2^ = .321) than for Jewish participants (F_1,67_ = 9.09, p = .004, η_p_^2^ = .12). Taken together, the findings indicate that the intervention effects reported earlier are quite robust to sex and ethnic affiliation.
